# A high-quality chromosome-level reference genome of *Pulsatilla chinensis*

**DOI:** 10.1038/s41597-025-06479-3

**Published:** 2025-12-24

**Authors:** Ting Wang, Chenhao Zhang, Xiao Ma, Yanhong Fu, Yinglong Xiang, Chengyan Wu, Shaoqin Shen, Zhuo Liu, Kejun Deng, Hao Lin, Xiaoming Song

**Affiliations:** 1 School of Life Science and Technology and Center for Informational Biology, University of Electronic Science and Technology of China, Chengdu, 610054 China; 2 School of Life Sciences/School of Basic Medical Sciences/Library, North China University of Science and Technology, Tangshan, 063210 China; 3 Chuzhou Integrated Traditional Chinese and Western Medicine Hospital Affiliated to Anhui University of Chinese Medicine, Chuzhou, 239000 China; 4 School of Physical Science and Technology, Key Laboratory of Magnetism and Magnetic Materials for Higher Education in Inner Mongolia Autonomous Region, Baotou Teachers’ College, Baotou, 014030 China

**Keywords:** Bioinformatics, Genome duplication, Genetics research

## Abstract

*Pulsatilla chinensis* (Bunge) Regel, a medicinal species in the genus *Pulsatilla* (Ranunculaceae), exhibits significant anti-inflammatory activity due to its abundant triterpenoid saponins, enhancing its medicinal value. All 48 known *Pulsatilla* species lack whole genome sequencing data, hindering research on the utilization of genetic resources in the genus and investigations into their biosynthetic mechanisms. Here, we present the first chromosome-level genome assembly of *P. chinensis*. The assembly spans 6,588.76 Mb with a contig N50 of 4.28 Mb. Using PacBio sequencing and Hi-C scaffolding, 99.32% of sequences were anchored onto eight chromosomes. We annotated 41,786 protein-coding genes through *ab initio* prediction, homology alignment, and transcriptome evidence, with 98.77% functionally annotated via NR, GO, and KEGG databases. This assembly not only facilitates studies on the molecular mechanisms and biosynthesis of bioactive compounds in *P. chinensis*, but also supports the further enrichment and utilization of germplasm resources across the Ranunculaceae.

## Background & Summary

*Pulsatilla chinensis* (Bunge) Regel (2n = 16), a plant of medicinal and ornamental value, derives its name from the distinctive white hairs covering its fruit (“Baitouweng” translates to “white-haired elder”). The genus *Pulsatilla* currently comprises 48 accepted species worldwide (*Plants of the World Online*; accessed 20 Oct 2025)^1^, which predominantly distributed across Asia, North America, and Europe. In China, 12 species are documented, primarily inhabiting northern and northeastern regions^2,3^. In traditional Chinese medicine (TCM), the dried rhizomes of *P. chinensis* are used to clear heat, detoxify, cool blood, and alleviate dysentery. They have also been employed to treat malaria and relieve muscle spasms. Modern pharmacological studies confirm that bioactive compounds isolated from *Pulsatilla* exhibit demonstrated therapeutic potential, including antitumor, anti-inflammatory, antibacterial, and antiviral effects.

Saponins are a class of secondary metabolites in plants and marine organisms, categorized into two major types: triterpenoid saponins and steroid saponins^4,5^. To date, triterpenoids, flavonoids, coumarins, and lignans have been isolated from *Pulsatilla chinensis*, with triterpenoid saponins identified as the primary medicalcomponents^6,7^. Given their efficacy, saponins represent promising lead compounds for developing natural product-derived drugs. The universal precursor of all triterpenes saponins is 2,3-oxidosqualene, synthesized through two distinct pathways: the cytosolic mevalonate (MVA) pathway and the methylerythritol phosphate (MEP) pathway. This precursor is subsequently cyclized by oxidosqualene cyclases (*OSCs*) into diverse triterpene and sterol scaffolds. The second diversification step in triterpene biosynthesis is primarily mediated by cytochrome P450 monooxygenases (*CYPs*) and UDP-glycosyltransferases (*UGTs*), which catalyze hydroxylation, oxidation, epoxidation, and glycosylation reactions^8^. A primary limitation in analyzing active compound biosynthesis in medicinal resources is the scarcity of high-quality genomic data. Advances in sequencing technologies have enabled significant progress in genomics, including successful genome assemblies for medicinal species such as taxol-producing *Taxus*^9^, triterpene-rich *Ganoderma lucidum*^10^ and aescin-synthesizing *Aesculus hippocastanum*^11^. Comparative genomics further facilitates exploration of biosynthetic pathways within related taxa. For instance, genome assemblies of *Saruma henryi* and *Aristolochia manshuriensis* elucidated the biosynthesis of benzylisoquinoline alkaloids (BIAs)^12^. These studies fully demonstrate the significant potential of genomics in elucidating complex biosynthetic processes and intricate metabolic regulatory pathways of medicinal plants, providing valuable insights for novel and functional natural product-derived drug discovery. Although the enzyme genes in triterpene synthesis in many plants have been functionally identified, the lack of genomic information has hindered the study of the synthesis pathway of saponins. This undoubtedly makes the biosynthesis process of saponins a problem, which further limits the research on its medicinal activity and the mining of its medicinal value. However, the genome of *P. chinensis* remains unreported, hindering understanding of its active compound biosynthesis and greatly limiting its further functional development and utilization.

*Pulsatilla chinensis* is a species within the genus *Pulsatilla* (Ranunculaceae). Although the Ranunculaceae is species-rich, whole-genome sequencing and assemblies remain scarce, and chromosome-level, high-quality assemblies are even rarer (GoaT database)^13^. To date, most available genomes in this family are concentrated in *Aquilegia* and *Coptis*^13–16^. Currently, no genome of any *Pulsatilla* species has been sequenced or reported. To address this gap, we performed chromosome-level whole-genome sequencing and assembly of *P. chinensis*, obtaining a high-quality genome spanning 6.59 Gb (6,588.76 Mb). To our knowledge, this work provides not only the first high-quality reference genome for *Pulsatilla* but also the largest reference genome reported so far in Ranunculaceae^13^.Collectively, these data provide critical support for elucidating the biosynthetic pathways of *P. chinensis* metabolites and establish a foundational resource for comparative and functional genomics across the Ranunculaceae family and Ranunculales order.

## Methods

### Plant materials and sequencing

We collected single three-year-old flowering *Pulsatilla chinensis* planted in Chuzhou Hospital of Integrated Traditional Chinese and Western Medicine, Chuzhou City, Anhui Province for genome sequence and assembly. The sample storage ID is SAMEA120389571^17^. The genome of *Pulsatilla chinensis* was sequenced using PacBio HiFi, Illumina, and Hi-C technologies. The leaves of freshly collected plants were taken to extract genomic DNA according to the CTAB method^18^. Total RNA was extracted from roots, leaves, and flowers using TRIzol reagent following standard protocols. All collected plant samples were immediately washed with ultrapure water, frozen in liquid nitrogen, and stored at −80 °C. The purity and concentration of genomic DNA were evaluated using a spectrophotometer NanoDrop 2000 (Thermo Fisher Scientific, USA) for the extracted nucleic acids. DNA integrity was detected using LabChip GX Touch HT (PerkinElmer, USA). Subsequently, DNA quantification was performed using Qubit 3.0 (Invitrogen, USA). For Illumina short-read sequencing, paired-end 150 bp (PE150) sequencing was performed on the Illumina NovaSeq X Plus platform, generating approximately 350.99 Gb (55.95 × coverage) of data subsequently used for genome survey analysis and assembly error correction. PacBio library construction utilized genomic DNA from the same sample, yielding approximately 197.18 Gb (31 × coverage) of HiFi data on the Revio platform (PacBio, USA). The Hi-C library was constructed via *in situ* Hi-C methodology involving cell cross-linking, endonuclease digestion, end repair, ligation, DNA purification and capture, followed by PE150 sequencing on the Illumina platform based on Sequencing by Synthesis (SBS) technology, which produced 755.83 Gb of high-quality data (Table 1).

**Table 1:** Statistics of sequencing data for *Pulsatilla chinensis* genome assembly.

Methodology	Platform	Total length (Gb)	Reads number
Illumina paired-end short reads	Illumina NovaSeq 6000	350.99	2,344,273,884
PacBio HiFi reads	PacBio Sequel	197.18	10,311,708
Hi-C reads	Illumina NovaSeq 6000	755.83	2,525,004,035

### Genome survey

**Fig. 1:**
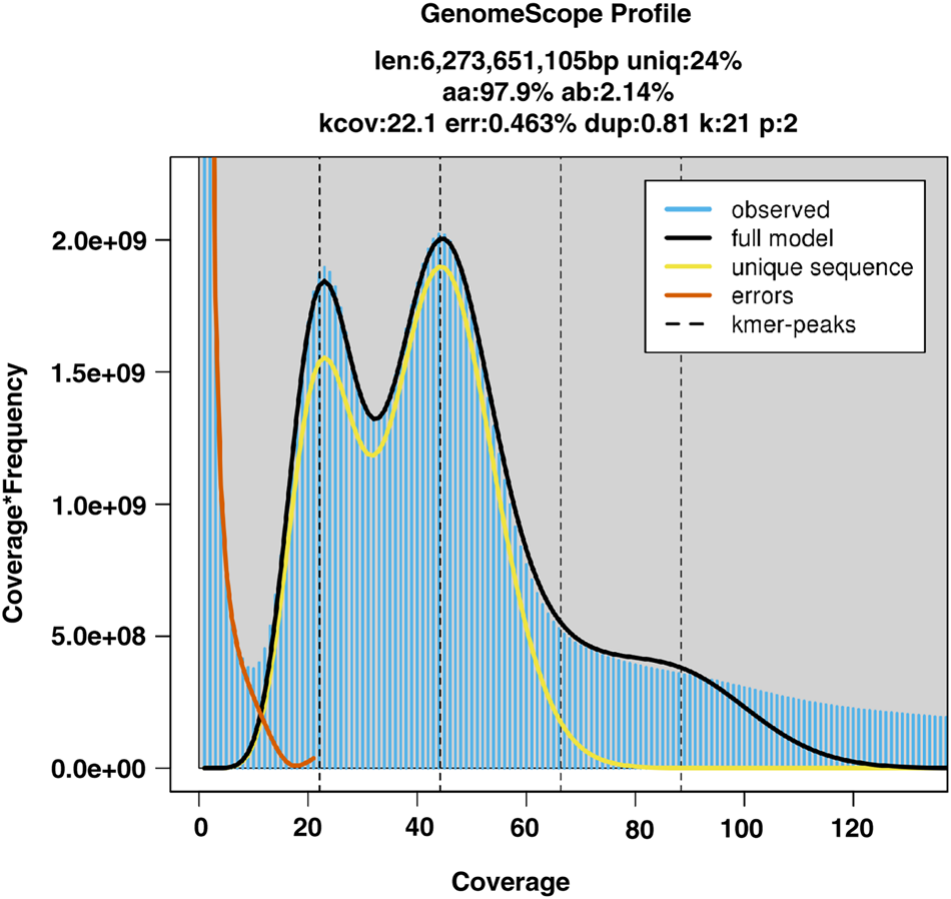
The survey plot of *Pulsatilla chinensis* genome estimation.

Illumina short reads were filtered using Fastp v0.21.0 with default parameters to remove adapter sequences, duplicates, and low-quality reads^19^. Quality assessment of the processed data showed Q20 and Q30 ratios exceeding 98.24% and 90.58%, respectively, with a GC content of approximately 40.34%. To assess potential DNA contamination, 10,000 randomly selected single-end reads from a 350 bp library were aligned against the NT database using BLAST^20^ (ncbi-blast + v2.2.29; parameters: -num_descriptions 100 -num_alignments 100 -evalue 1e-05), confirming no contamination. For organellar DNA evaluation, Illumina libraries were aligned to plastid sequences via SOAP v2.21^21^(parameters: -m 260 -x 440), revealing that both paired-end and single-end mapped reads were below the empirical threshold of 5%, indicating minimal organellar DNA interference with survey analysis. Genome size and heterozygosity were estimated through k-mer analysis using Jellyfish v2.1.4^22^ (parameters: -h 1000000000) and GenomeScope v2.0^23^ (parameters: -k 21 -p 2 -m 10000000) with 350 bp library data (Fig. 1). This predicted a genome size of 6.27 Gb, repeat content of 75.98%, and heterozygosity rate of 2.14% (Table 2). Flow cytometry further validated the genome size at 6.64 Gb, confirming *P. chinensis* possesses a large, highly heterozygous complex genome.

**Table 2:** K-mer analysis of the *Pulsatilla chinensis* genome.

Terms	Value
K-mer size	21
K-mer depth	22.1
Estimated genome size (Gb)	6.27
Heterozygous ratio (%)	2.14
Repeat (%)	75.98

“K-mer depth” values represent the peak values of the k-mer frequency distribution.

### *De novo* genome assembly

**Fig. 2:**
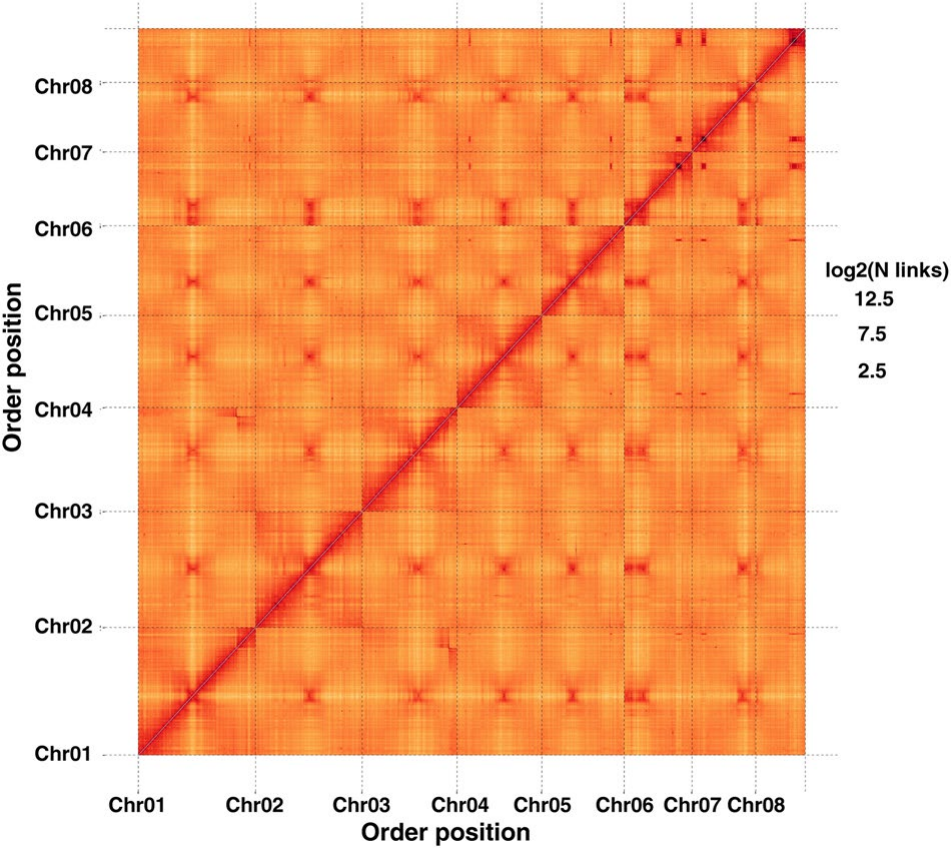
The Hi-C contact map of *Pulsatilla chinensis* genome assembly.

PacBio HiFi sequencing generated approximately 197.18 Gb of clean data with an average read length of 19.12 Kb. After data filtering, the total length of the genomic Contig sequence is about 7.29 Gb and the Contig N50 is about 3.83 Mb by splicing by Hifiasm v0.16.0^24^ (parameters: -l 2 -n 4) (Table 3). Integration of 548.17 Gb of DNA sequences from both Illumina and PacBio platforms yielded a preliminary *Pulsatilla chinensis* assembly spanning 6.59 Gb, with a contig N50 of 4.28 Mb. A total of 752.98 Gb of Hi-C sequencing data was obtained to enhance the quality of the *P. chinensis* genome assembly. The data were processed using HiC-Pro v2.10.0^25^ for filtering and evaluation and then aligned to the draft genome using BWA v0.7.17-r1188^26^ to assess library quality. Chromosome-level scaffolding was achieved using LACHESIS (version 20171221)^27^ for clustering, ordering, and orienting contigs, followed by manual curation guided by Hi-C contact maps (Fig. 2). The final genome assembly spanned about 6,588.76 Mb, with contig N50 and scaffold N50 values reaching 4.28 Mb and 813.62 Mb, respectively (Table 4). Of this, 6,543.97 Mb (99.32% of the assembly) was anchored to eight chromosomes (Table 5), supported by a read mapping rate exceeding 99.38%, confirming high assembly completeness.

**Table 3:** Statistics of Hifiasm Assembly Results.

Metrics	Value
Contig Number (>1 kb)	4,396
Contig Length (bp)	7,292,487,671
Contig Max (bp)	48,215,453
Contig N50 (bp)	3,833,233
Contig N90 (bp)	868,204
GC content (%)	40.59

**Table 4:** Statistics of genome assembly results of *Pulsatilla. chinensis* by Hi-C technology.

Metrics	Value
Total length (Gb)	6.59
Contig max (bp)	48,215,453
Contig number	2,670
Contig N50 (bp)	4,277,949
Contig N90 (bp)	1,317,962
GC content (%)	40.52
Total mapped length (Gb)	6.54
Mapping rate (%)	99.32

**Table 5:** The assembled length and cluster number of each chromosome of *Pulsatilla chinensis* genome.

Group	Cluster Num	Cluster Len (bp)	Order Num	Order Len (bp)
Chr01	443	1,153,917,847	389	1,126,958,398
Chr02	413	1,029,122,201	359	1,021,004,186
Chr03	345	922,214,884	305	913,659,524
Chr04	296	836,201,734	252	813,590,232
Chr05	355	813,025,120	308	793,998,544
Chr06	216	666,735,353	178	654,318,483
Chr07	249	620,589,196	207	614,035,171
Chr08	213	502,167,257	180	477,481,469
Total (Ratio %)	2,530(94.76)	6,543,973,592(99.32)	2,178(86.09)	6,415,046,007(98.03)

### Repeat elements

**Fig. 3:**
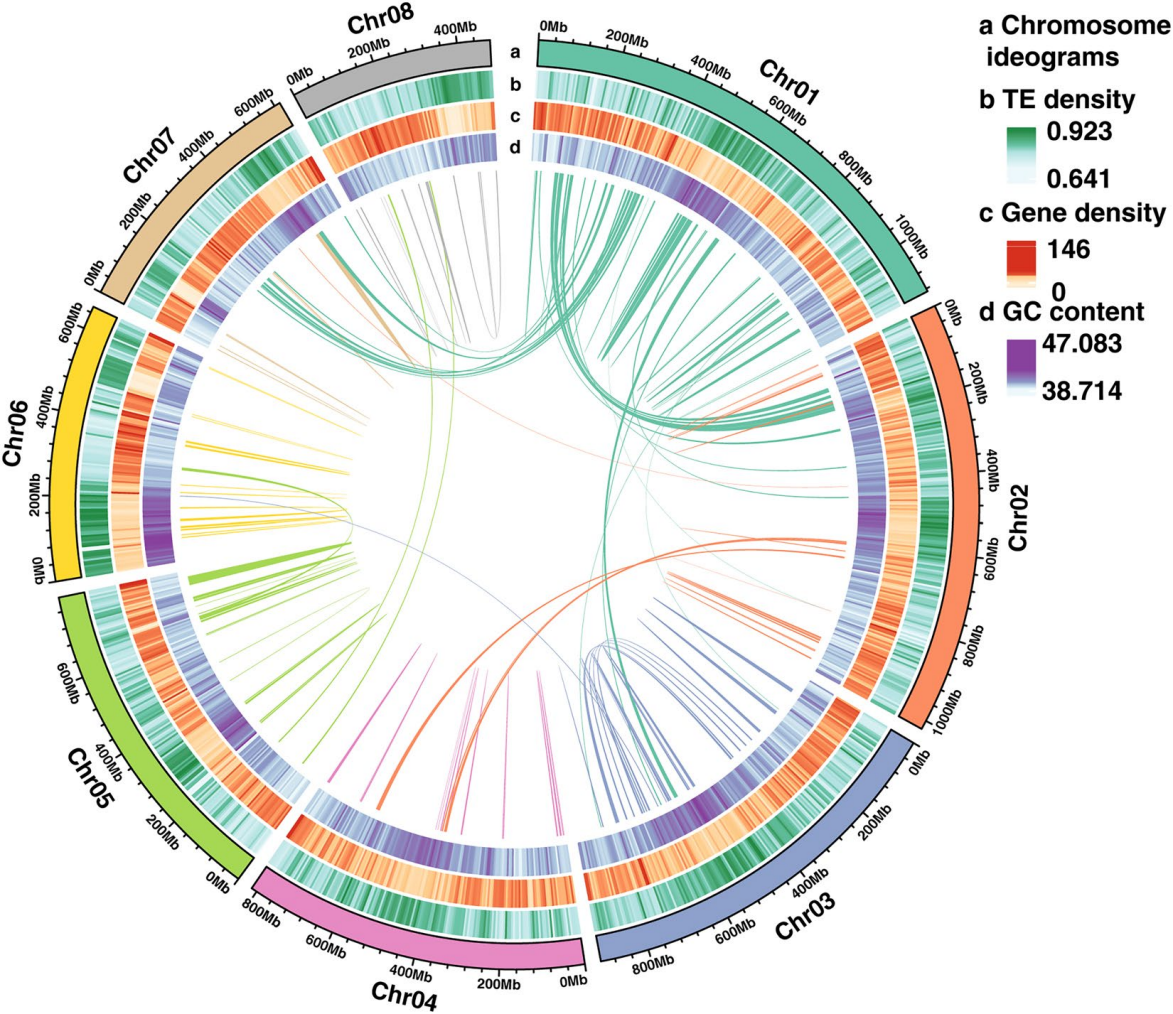
The distribution of transposable element, gene density, and GC content on each chromosome in *Pulsatilla chinensis*.

Repeated sequences in the *Pulsatilla chinensis* genome primarily comprise tandem repeats and interspersed repeats, with the latter dominated by transposable elements (TEs). To annotate these repeats, we employed a multi-step approach: First, de novo prediction was performed using RepeatModeler2 v2.0.1^28^, integrating outputs from RECON v1.0.8^29^ and RepeatScout v1.0.6^30^, followed by classification via RepeatClassifier with the Dfam v3.5 database. Second, LTR retrotransposons were specifically identified using LTR_retriever v2.9.0^31^, which consolidated predictions from LTRharvest v1.5.10^32^ and LTR_FINDER v1.07^33^. The combined de novo predictions and known repeats were merged and deduplicated to generate a species-specific repeat library. This library was then utilized by RepeatMasker v4.1.2^33^ for genome-wide TE annotation. Tandem repeats were detected using MIcroSAtellite identification tool (MISA v2.1)^34^ and Tandem Repeat Finder^35^(TRF v4.09; parameters: 2 7 7 80 10 50 500 -d -h). Collectively, repetitive sequences constituted 81.09% of the genome (Fig. 3), with long terminal repeats (LTRs) representing the majority (4,903.14 Mb, 74.42%) (Table 6). Among LTRs, Gypsy-type elements were predominant, accounting for 48.84% of the total genome.

**Table 6:** The statistics of the repeat sequence classification in *Pulsatilla chinensis* genome.

Type	Number	Length (bp)	Rate (%)
TE (transposable elements)	4,066,698	5,342,772,551	81.09
TRs (tandem repeats)	2,636,203	385,147,844	5.85

### Gene prediction and function annotation

Gene prediction in *Pulsatilla chinensis* integrated homology-based, *de novo* and transcriptome-based methods. *De novo* prediction employed Augustus v3.1.0^36^ and SNAP v38925^37^, while homology-based prediction utilized GeMoMa v1.7^38^ with related species (*Coptis chinensis*^39^, *Corydalis tomentella*^40^, *Arabidopsis thalian*^41^, *Aquilegia coerulea*^42^) (Table 7). Transcriptome-based prediction utilized transcripts assembled through two distinct strategies. One employed Hisat v2.1.0^43^ and StringTie v2.1.4^44^ for transcript assembly followed by gene prediction using GeneMarkS-T v5.1^45^. The other generated transcripts via Trinity v2.11^46^ assembly and subsequently predicting genes with PASA v2.4.1^47^. EVM v1.1.1^48^ consolidated predictions from all three methods, with final gene models refined by PASA v2.4.1, yielding 41,786 predicted genes (Table 8). Of these, 41,271 genes (98.77%) were functionally annotated against NR, eggNOG, GO, KEGG, TrEMBL, KOG, SWISS-PROT and Pfam databases (Table 9).

**Table 7:** Genomic data information for homologous predictive species.

Species	Database	retrieval date	Accession number
*Coptis chinensis*^39^	NCBI	2023-12	GCA_015680905.1
*Corydalis tomentella*^40^	GWH	2023-12	GWHAORS00000000
*Arabidopsis thalian*^41^	NCBI	2023-12	GCA_028009825.2
*Aquilegia coerulea*^42^	NCBI	2023-12	GCA_002738505.1

**Table 8:** The statistical of gene prediction in *Pulsatilla chinensis* genome.

Method	Software	Species	Gene number
Ab initio	Augusts	—	43,198
	SNAP	—	128,986
Homology-based	GeMoMa	*A. coerulea*	40,635
		*A. thaliana*	33,054
		*C. chinensis*	49,923
		*C. tomentella*	53,774
RNAseq	GeneMarkS-T	—	22,945
	PASA	—	17,548
Integration	EVM	—	41,786

**Table 9:** Statistics of gene functional annotations in *Pulsatilla chinensis* genome.

Annotated Database	Annotated Number	Annotated Ratio (%)
GO	34,720	83.09
KEGG	34,115	81.64
KOG	26,125	62.52
Pfam	24,983	59.79
Swissprot	34,721	83.09
TrEMBL	41,227	98.66
EggNOG	36,335	86.95
Nr	40,901	97.88
All	41,271	98.77

Non-coding RNAs (ncRNAs) are defined as RNA molecules that do not encode proteins, encompassing functionally diverse types including microRNAs, rRNAs, and tRNAs. Specific tools were employed to predict distinct ncRNA types based on their structural features. TRNAscan-SE v1.3.1^49^ identified tRNAs. Barrnap v0.9^50^ predicted rRNAs. And Infernal v1.1^51^ with the Rfam v14.5^52^ database detected miRNAs, snoRNAs, and snRNAs. Collectively, this analysis predicted 9,398 rRNAs, 138 miRNAs, 3,093 tRNAs, 937 snRNAs, and 475 snoRNAs (Fig. 3).

## Data Records

All raw sequencing reads and assembly and annotation files generated in this study have been deposited in the NCBI Sequence Read Archive (SRA) under the study ERP182249 (https://identifiers.org/ncbi/insdc.sra:ERP182249^53^). This study includes Illumina paired-end genomic reads, Hi-C reads, PacBio HiFi (cells 1–7), Iso-Seq reads, RNA-seq reads and annotation files. Individual run accessions (ERR) are provided in Table 10. The study is registered under BioProject PRJEB100803^54^. The genome assembly have been deposited in the ENA/NCBI database under the accession number GCA_977018565.1^55^. Annotation data is available in both the figshare [10.6084/m9.figshare.30521519]^56^ and ENA databases.

**Table 10:** Inclusion and access to information from all datasets obtained in this study.

Data type	Database	Accession/DOI	Version	file types	release date	MD5 checksums
BioProject	NCBI	PRJEB100803^54^	\	\	2025-11-4	\
BioSample	NCBI	SAMEA120389571^17^	\	\	2025-11-4	\
Illumina paired-end short reads	NCBI	ERR15811584^59^	\	fastq	2025-11-4	See MD5sums_manifest (10.6084/m9.figshare.30521519)
PacBio HiFi reads (cell1-cell7)	NCBI	ERR15749367^60^ ERR15755657^61^ ERR15757035^62^ ERR15761646^63^ ERR15764838^64^ ERR15765418^65^ ERR15765427^66^	\	bam	2025-11-4	See MD5sums_manifest (10.6084/m9.figshare.30521519)
Hi-C reads	NCBI	ERR15807318^67^	\	fastq	2025-11-4	See MD5sums_manifest (10.6084/m9.figshare.30521519)
RNA-seq reads	NCBI	ERR15729319^68^ ERR15811596^69^ ERR15811690^70^	\	fastq	2025-11-4	See MD5sums_manifest (10.6084/m9.figshare.30521519)
Iso-Seq reads	NCBI	ERR15813363^71^ ERR15813894^72^ ERR15814006^73^	\	bam	2025-11-4	See MD5sums_manifest (10.6084/m9.figshare.30521519)
Genome assembly	NCBI/ENA	GCA_977018565.1^55^	v1	fasta	2025-11-9	See MD5sums_manifest (10.6084/m9.figshare.30521519)
Annotation files	Figshare/ENA	10.6084/m9.figshare.30521519/ERP182249	v1.0	gff/fasta	2025-11-4	See MD5sums_manifest (10.6084/m9.figshare.30521519)

## Technical Validation

### Evaluation of the assembled genome

To evaluate the final genome assembly quality, we conducted comprehensive assessments using sequencing data. For Illumina data, paired-end reads were aligned to the draft genome via BWA v7.17-r1188 (aln mode, default parameters), achieving an alignment rate of 99.38%. HiFi reads were mapped to the assembled genome using Minimap2 v2.14-r883, yielding a 99.83% alignment rate, collectively confirming assembly completeness. Two additional methods were employed. CEGMA v2.5^57^ analysis identified 401 out of 458 conserved eukaryotic core genes (87.55%) through tBLASTn, GeneWise v2.4.1, and GeneID. BUSCO v5.2.1^58^ assessment against the embryophyta database (OrthoDB v10) revealed 90.46% completeness (Table 11). For Hi-C validation, the chromosome-level assembly was partitioned into 5 Mb bins, with interaction intensities visualized via heatmaps. The genome assembly of *Pulsatilla chinensis* was clearly divided into eight chromosomal clusters. Within each cluster, interaction intensities at diagonal positions exceeded those at off-diagonal positions, indicating stronger proximal interactions and weaker distal signals in the Hi-C-based chromosome assembly. This pattern aligns with Hi-C scaffolding principles, validating the high quality of the genome assembly.

**Table 11:** Completeness of the assembled genomes and gene prediction evaluation.

Method	Total genes in database	Number of genes present in assembly/prediction	Ratio (%)
CEGMA	458	401	87.55
BUSCO	1614	1460	90.46
**Gene prediction evalution**			
BUSCO	1614	1549	95.42

### Coding gene prediction and evaluation

**Fig. 4:**
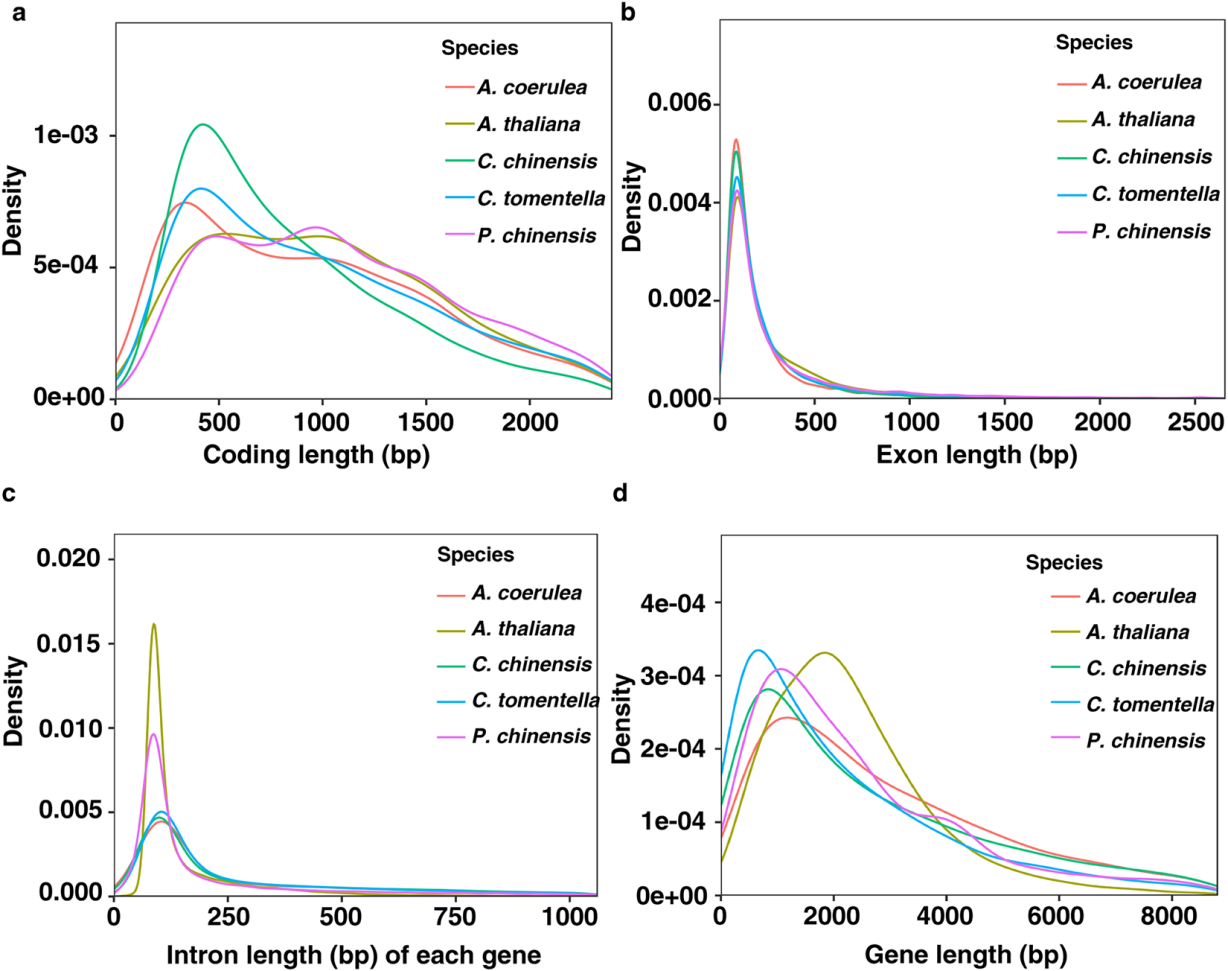
The distribution map of gene characteristics of each species in the prediction and evaluation of homologous species.

EVM v1.1.1 integrated genes were quantified based on support from three prediction methods. Most genes were supported by at least two methods, indicating high prediction reliability. Genomic features—including gene length, exon length and count, intron length and count, and coding sequence (CDS) length—were compared across phylogenetically close speciesto assess prediction quality (Fig. 4). Using BUSCO v5.2.2 with the embryophyta database (1,614 conserved core genes), 95.42% of BUSCO genes were present in the predicted gene set, confirming high completeness of gene prediction (Table 11).

## Data Availability

All raw reads and genome assembly result can be obtained from NCBI database. The specific accession as it is described in the Data Record section and Table 10. Related annotation files can be accessed from the Figshare databases (10.6084/m9.figshare.30521519.v1) and ENA database.

## Code availability

This study did not use any specific code. All data analyses were performed with standard bioinformatic tools, and the specific parameters and software versions are detailed in the Methods section. For tools without specified parameters, default settings recommended by the developers were applied. All software and codes utilized in this study are publicly accessible.

## References

[1] *Plants of the World Online*. http://powo.science.kew.org/taxon/urn:lsid:ipni.org:names:325533-2 (2025).

[2] Gao, N., Liu, T., Yang, Y. & Xu, L. Research progress on pentacyclic triterpenoid constituents and pharmacological effects in Pulsatilla species in China. *Chin. Arch. Tradit. Chin. Med.* **1**, 17 (2025).

[3] Zhang, T.-T., Zhang, S.-M., Xu, L. & Kang, T.-G. Pulsatilla saxatilis (Ranunculaceae), a new species from north-east China. *Phytotaxa* **539**, 195–202 (2022).

[4] Abe, I., Rohmer, M. & Prestwich, G. D. Enzymatic cyclization of squalene and oxidosqualene to sterols and triterpenes. *Chem. Rev.* **93**, 2189–2206 (1993).

[5] Vincken, J.-P., Heng, L., de Groot, A. & Gruppen, H. Saponins, classification and occurrence in the plant kingdom. *Phytochemistry* **68**, 275–297 (2007). 17141815 10.1016/j.phytochem.2006.10.008

[6] Leng, D. A Comprehensive Review on Botany, Phytochemistry, Traditional Uses, Pharmacology, Analytical Methods, Processing Methods, Pharmacokinetics and Toxicity of Pulsatilla chinensis. *Altern. Ther. Health Med.* **30**, 374–380 (2024). 37793336

[7] Zhong, J., Tan, L., Chen, M. & He, C. Pharmacological activities and molecular mechanisms of Pulsatilla saponins. *Chin. Med.* **17**, 59 (2022). 35606807 10.1186/s13020-022-00613-8 PMC9125917

[8] Reed, J. *et al*. Elucidation of the pathway for biosynthesis of saponin adjuvants from the soapbark tree. *Science* **379**, 1252–1264 (2023). 36952412 10.1126/science.adf3727

[9] Li, Z. *et al*. Phased high-quality genome of the gymnosperm Himalayan Yew assists in paclitaxel pathway exploration. *GigaScience* **14**, giaf026 (2025). 40184434 10.1093/gigascience/giaf026 PMC11970372

[10] Jiang, N. *et al*. Massive genome investigations reveal insights of prevalent introgression for environmental adaptation and triterpene biosynthesis in Ganoderma. *Mol. Ecol. Resour.* **25**, e13718 (2025). 36214617 10.1111/1755-0998.13718

[11] Sun, W. *et al*. Characterization of the horse chestnut genome reveals the evolution of aescin and aesculin biosynthesis. *Nat. Commun.* **14**, 6470 (2023). 37833361 10.1038/s41467-023-42253-y PMC10576086

[12] Hu, Y. *et al*. Evolutionary history of magnoliid genomes and benzylisoquinoline alkaloid biosynthesis. *Nat. Commun.* **16**, 4039 (2025). 40301376 10.1038/s41467-025-59343-8 PMC12041406

[13] Challis, R., Kumar, S., Sotero-Caio, C., Brown, M. & Blaxter, M. Genomes on a Tree (GoaT): A versatile, scalable search engine for genomic and sequencing project metadata across the eukaryotic tree of life. *Wellcome Open Res.* **8**, 24 (2023). 36864925 10.12688/wellcomeopenres.18658.1 PMC9971660

[14] Liu, Y. *et al*. Analysis of the Coptis chinensis genome reveals the diversification of protoberberine-type alkaloids. *Nat. Commun.* **12**, 3276 (2021). 34078898 10.1038/s41467-021-23611-0 PMC8172641

[15] Mokhtar, M. M., Abd-Elhalim, H. M. & El Allali, A. A large-scale assessment of the quality of plant genome assemblies using the LTR assembly index. *Aob Plants* **15**, plad015 (2023). 37197714 10.1093/aobpla/plad015 PMC10184434

[16] Xie, J. *et al*. A chromosome-scale reference genome of Aquilegia oxysepala var. *kansuensis. Hortic. Res.* **7**, 113 (2020). 32637141 10.1038/s41438-020-0328-y PMC7326910

[17] *NCBI BioSample* https://www.ncbi.nlm.nih.gov/biosample/SAMEA120389571 (2025).

[18] Porebski, S., Bailey, L. G. & Baum, B. R. Modification of a CTAB DNA extraction protocol for plants containing high polysaccharide and polyphenol components. *Plant Mol. Biol. Report*. **15** (1997).

[19] Chen, S. Ultrafast one-pass FASTQ data preprocessing, quality control, and deduplication using fastp. *iMeta* **2**, e107 (2023). 38868435 10.1002/imt2.107 PMC10989850

[20] Altschul, S. F., Gish, W., Miller, W., Myers, E. W. & Lipman, D. J. Basic local alignment search tool. *J. Mol. Biol.* **215**, 403–410 (1990). 2231712 10.1016/S0022-2836(05)80360-2

[21] Li, R., Li, Y., Kristiansen, K. & Wang, J. SOAP: short oligonucleotide alignment program. *Bioinformatics* **24**, 713–714 (2008). 18227114 10.1093/bioinformatics/btn025

[22] Marçais, G. & Kingsford, C. A fast, lock-free approach for efficient parallel counting of occurrences of k-mers. *Bioinformatics* **27**, 764–770 (2011). 21217122 10.1093/bioinformatics/btr011 PMC3051319

[23] Ranallo-Benavidez, T. R., Jaron, K. S. & Schatz, M. C. GenomeScope 2.0 and Smudgeplot for reference-free profiling of polyploid genomes. *Nat. Commun.* **11**, 1432 (2020). 32188846 10.1038/s41467-020-14998-3 PMC7080791

[24] Cheng, H., Concepcion, G. T., Feng, X., Zhang, H. & Li, H. Haplotype-resolved de novo assembly with phased assembly graphs. *Nat. Methods* **18**, 170–175 (2021). 33526886 10.1038/s41592-020-01056-5 PMC7961889

[25] Servant, N. *et al*. HiC-Pro: an optimized and flexible pipeline for Hi-C data processing. *Genome Biology* **16**(1), 1–11 (2015). 26619908 10.1186/s13059-015-0831-x PMC4665391

[26] Li, H. & Durbin, R. Fast and accurate short read alignment with Burrows–Wheeler transform. *Bioinformatics* **25**, 1754–1760 (2009). 19451168 10.1093/bioinformatics/btp324 PMC2705234

[27] Burton, J. N. *et al*. Chromosome-scale scaffolding of de novo genome assemblies based on chromatin interactions. *Nat. Biotechnol.* **31**, 1119–1125 (2013). 24185095 10.1038/nbt.2727 PMC4117202

[28] Flynn, J. M. *et al*. RepeatModeler2 for automated genomic discovery of transposable element families. *Proc. Natl. Acad. Sci. USA.* **117**, 9451–9457 (2020). 32300014 10.1073/pnas.1921046117 PMC7196820

[29] Bao, Z. & Eddy, S. R. Automated de novo identification of repeat sequence families in sequenced genomes. *Genome Res.* **12**, 1269–1276 (2002). 12176934 10.1101/gr.88502 PMC186642

[30] Price, A. L., Jones, N. C. & Pevzner, P. A. De novo identification of repeat families in large genomes. *Bioinforma. Oxf. Engl.* **21**(Suppl 1), i351–358 (2005). 10.1093/bioinformatics/bti1018 15961478

[31] Ou, S. & Jiang, N. LTR_retriever: A Highly Accurate and Sensitive Program for Identification of Long Terminal Repeat Retrotransposons. *Plant Physiol.* **176**, 1410–1422 (2018). 29233850 10.1104/pp.17.01310 PMC5813529

[32] Ellinghaus, D., Kurtz, S. & Willhoeft, U. LTRharvest, an efficient and flexible software for de novo detection of LTR retrotransposons. *BMC Bioinformatics* **9**, 18 (2008). 18194517 10.1186/1471-2105-9-18 PMC2253517

[33] Xu, Z. & Wang, H. LTR_FINDER: an efficient tool for the prediction of full-length LTR retrotransposons. *Nucleic Acids Res.* **35**, W265–W268 (2007). 17485477 10.1093/nar/gkm286 PMC1933203

[34] Beier, S., Thiel, T., Münch, T., Scholz, U. & Mascher, M. MISA-web: a web server for microsatellite prediction. *Bioinforma. Oxf. Engl.* **33**, 2583–2585 (2017). 10.1093/bioinformatics/btx198 PMC5870701 28398459

[35] Benson, G. Tandem repeats finder: a program to analyze DNA sequences. *Nucleic Acids Res.* **27**, 573–580 (1999). 9862982 10.1093/nar/27.2.573 PMC148217

[36] Stanke, M., Diekhans, M., Baertsch, R. & Haussler, D. Using native and syntenically mapped cDNA alignments to improve de novo gene finding. *Bioinforma. Oxf. Engl.* **24**, 637–644 (2008). 10.1093/bioinformatics/btn013 18218656

[37] Korf, I. Gene finding in novel genomes. *BMC Bioinformatics* **5**, 59 (2004). 15144565 10.1186/1471-2105-5-59 PMC421630

[38] Keilwagen, J. *et al*. Using intron position conservation for homology-based gene prediction. *Nucleic Acids Res.* **44**, e89 (2016). 26893356 10.1093/nar/gkw092 PMC4872089

[39] *NCBI GenBank.* https://identifiers.org/ncbi/insdc.gca:GCA_015680905.1/ (2023).

[40] *CNCB Genome Warehouse*. https://ngdc.cncb.ac.cn/gwh/Assembly/10358/show/ GWHAORS00000000 (2023).

[41] *NCBI GenBank.* https://identifiers.org/ncbi/insdc.gca:GCA_000001735.1 (2023).

[42] *NCBI GenBank.* https://identifiers.org/ncbi/insdc.gca:GCA_002738505.1/ (2023).

[43] Kim, D., Langmead, B. & Salzberg, S. L. HISAT: a fast spliced aligner with low memory requirements. *Nat. Methods* **12**, 357–360 (2015). 25751142 10.1038/nmeth.3317 PMC4655817

[44] Pertea, M. *et al*. StringTie enables improved reconstruction of a transcriptome from RNA-seq reads. *Nat. Biotechnol.* **33**, 290–295 (2015). 25690850 10.1038/nbt.3122 PMC4643835

[45] Tang, S., Lomsadze, A. & Borodovsky, M. Identification of protein coding regions in RNA transcripts. *Nucleic Acids Res.* **43**, e78 (2015). 25870408 10.1093/nar/gkv227 PMC4499116

[46] Grabherr, M. G. *et al*. Trinity: reconstructing a full-length transcriptome without a genome from RNA-Seq data. *Nat. Biotechnol.* **29**, 644–652 (2011). 21572440 10.1038/nbt.1883 PMC3571712

[47] Haas, B. J. *et al*. Improving the Arabidopsis genome annotation using maximal transcript alignment assemblies. *Nucleic Acids Res.* **31**, 5654–5666 (2003). 14500829 10.1093/nar/gkg770 PMC206470

[48] Haas, B. J. *et al*. Automated eukaryotic gene structure annotation using EVidenceModeler and the Program to Assemble Spliced Alignments. *Genome Biol.* **9**, R7 (2008). 18190707 10.1186/gb-2008-9-1-r7 PMC2395244

[49] Lowe, T. M. & Eddy, S. R. tRNAscan-SE: a program for improved detection of transfer RNA genes in genomic sequence. *Nucleic Acids Res.* **25**, 955–964 (1997). 9023104 10.1093/nar/25.5.955 PMC146525

[50] Loman, T. A Novel Method for Predicting Ribosomal RNA Genes in Prokaryotic Genomes. http://lup.lub.lu.se/student-papers/record/8914064 (2017).

[51] Nawrocki, E. P. & Eddy, S. R. Infernal 1.1: 100-fold faster RNA homology searches. *Bioinformatics* **29**, 2933–2935 (2013). 24008419 10.1093/bioinformatics/btt509 PMC3810854

[52] Griffiths-Jones, S. *et al*. Rfam: annotating non-coding RNAs in complete genomes. *Nucleic Acids Res.* **33**, D121–D124 (2005). 15608160 10.1093/nar/gki081 PMC540035

[53] *NCBI Sequence Read Archive* https://identifiers.org/ncbi/insdc.sra:ERP182249 (2025).

[54] *NCBI BioProject* https://www.ncbi.nlm.nih.gov/bioproject/PRJEB100803 (2025).

[55] *NCBI GenBank.* https://identifiers.org/ncbi/insdc.gca:GCA_977018565.1 (2025).

[56] Wang, ting Genome annotation files of Pulsatilla chinensis. *figshare*10.6084/m9.figshare.30521519.v1 (2025).

[57] Parra, G., Bradnam, K. & Korf, I. CEGMA: a pipeline to accurately annotate core genes in eukaryotic genomes. *Bioinforma. Oxf. Engl.* **23**, 1061–1067 (2007). 10.1093/bioinformatics/btm071 17332020

[58] Simão, F. A., Waterhouse, R. M., Ioannidis, P., Kriventseva, E. V. & Zdobnov, E. M. BUSCO: assessing genome assembly and annotation completeness with single-copy orthologs. *Bioinforma. Oxf. Engl.* **31**, 3210–3212 (2015). 10.1093/bioinformatics/btv351 26059717

[59] *NCBI Sequence Read Archive* http://identifiers.org/ena.embl:ERR15811584 (2025).

[60] *NCBI Sequence Read Archive* http://identifiers.org/ena.embl:ERR15749367 (2025).

[61] *NCBI Sequence Read Archive* http://identifiers.org/ena.embl:ERR15755657 (2025).

[62] *NCBI Sequence Read Archive* http://identifiers.org/ena.embl:ERR15757035 (2025).

[63] *NCBI Sequence Read Archive* http://identifiers.org/ena.embl:ERR15761646 (2025).

[64] *NCBI Sequence Read Archive* http://identifiers.org/ena.embl:ERR15764838 (2025).

[65] *NCBI Sequence Read Archive* http://identifiers.org/ena.embl:ERR15765418 (2025).

[66] *NCBI Sequence Read Archive* http://identifiers.org/ena.embl:ERR15765427 (2025).

[67] *NCBI Sequence Read Archive* http://identifiers.org/ena.embl:ERR15807318 (2025).

[68] *NCBI Sequence Read Archive* http://identifiers.org/ena.embl:ERR15729319 (2025).

[69] *NCBI Sequence Read Archive* http://identifiers.org/ena.embl:ERR15811596 (2025).

[70] *NCBI Sequence Read Archive* http://identifiers.org/ena.embl:ERR15811690 (2025).

[71] *NCBI Sequence Read Archive* http://identifiers.org/ena.embl:ERR15813363 (2025).

[72] *NCBI Sequence Read Archive* http://identifiers.org/ena.embl:ERR15813894 (2025).

[73] *NCBI Sequence Read Archive* http://identifiers.org/ena.embl:ERR15814006 (2025).

